# The Role of Microbiota in Type 1 Diabetes: Insights into Dysbiosis and Immune Interactions

**DOI:** 10.3390/nu18121904

**Published:** 2026-06-12

**Authors:** Ancuta Lupu, Emil Anton, Maria Oana Sasaran, Irina Tarnita, Ileana Ioniuc, Tania Elena Rusu, Stefana Moisa, Elena Tarca, Lacramioara Ionela Butnariu, Elena Cristina Mitrofan, Alin Horatiu Nedelcu, Sorana Caterina Anton, Anton Knieling, Ionela Daniela Morariu, Vasile Valeriu Lupu

**Affiliations:** 1 Grigore T. Popa University of Medicine and Pharmacy, 700115 Iasi, Romania; ancuta.ignat1@umfiasi.ro (A.L.); emil.anton@umfiasi.ro (E.A.); ileana.ioniuc@umfiasi.ro (I.I.); stefana-maria.moisa@umfiasi.ro (S.M.); tarca.elena@umfiasi.ro (E.T.); ionela.butnariu@umfiasi.ro (L.I.B.); alin.nedelcu@umfiasi.ro (A.H.N.); sorana.anton@umfiasi.ro (S.C.A.); anton.knieling@umfiasi.ro (A.K.); ionela.morariu@umfiasi.ro (I.D.M.); vasile.lupu@umfiasi.ro (V.V.L.); 2 Pediatrics, “George Emil Palade” University of Medicine, Pharmacy, Science and Technology, 540142 Targu Mures, Romania; oanam93@yahoo.com; 3 Regina Maria Healthcare Campus, 700023 Iasi, Romania; taniaelenarusu@gmail.com; 4 Clinic of Pulmonary Disease, 700115 Iasi, Romania; criselend@yahoo.com

**Keywords:** microbiome, dysbiosis, diabetes type 1, children

## Abstract

Type 1 Diabetes (T1D) is a complex autoimmune disorder characterized by immune-mediated destruction of pancreatic β cells, driven by genetic susceptibility and modulated by environmental factors, notably the gut microbiome. Dysbiosis, manifested as reduced microbial diversity, perturbations in the Firmicutes/Bacteroidetes ratio, and compromised short-chain fatty acid production, contributes to T1D pathogenesis through mechanisms involving immune system dysregulation and heightened intestinal permeability. Emerging evidence indicates a relationship between the gut and oral microbiomes, as well as the potential influence of the virome and mycobiome. This narrative review synthesizes the current literature on the intricate interplay between the gut microbial ecosystem, the host immune response, and the development of T1D, highlighting the potential for targeted microbiome-based interventions to ameliorate disease progression. A more nuanced understanding of these multi-kingdom interactions is essential for developing precise therapeutic strategies to prevent or delay T1D onset and to improve patient outcomes through restoration of immune tolerance and gut homeostasis.

## 1. Introduction

Type 1 Diabetes (T1D) is an autoimmune disorder characterized by the immune-mediated destruction of pancreatic islet β-cells, largely driven by autoreactive T lymphocytes, resulting in insufficient insulin synthesis. Its incidence has increased in developed countries over recent decades [1,2,3]. Disruptions in gut microbial balance, or dysbiosis, can increase intestinal permeability, alter the production of short-chain fatty acids (SCFAs), and provoke atypical immune responses that may contribute to T1D progression [4]. Additionally, the oral microbiome plays a crucial role in maintaining health, serving as the initial barrier against pathogenic microorganisms. Oral dysbiosis is linked to persistent inflammation and periodontal disease, both of which are common in individuals with T1D and can heighten systemic inflammatory states, complicating disease management. The autoimmune destruction of β cells in T1D is characterized by the generation of specific autoantibodies, often present months or years before clinical symptoms emerge—a phenomenon called seroconversion. Nonetheless, autoantibodies are absent in approximately 10–30% of cases, leaving the underlying mechanisms of β cell loss in these patients less well understood [5].

In recent years, the microbiota of the gastrointestinal tract has gained recognition as a key environmental factor influencing T1D onset. The human gut hosts around 100 trillion microorganisms—an amount ten times greater than the number of human cells [6]. Most of these microbes belong to just four main phyla: *Firmicutes*, *Bacteroidetes*, *Actinobacteria*, and *Proteobacteria* [7,8]. The intestinal microbiome is often described as a “hidden organ” [9] because of its diverse roles—including defending against infections [10], aiding in energy harvest [11], preserving the integrity of the gut lining [12,13], and modulating immune responses [14].

Since the mid-20th century, the prevalence of T1D has increased markedly across various industrialized nations. For example, in Finland, the incidence among children under 15 has escalated from 12 to 65 new cases per 100,000 annually over the past fifty years [15]. This rapid rise cannot be fully explained by genetic factors alone, implying that interactions between environmental influences—particularly those characteristic of Western lifestyles—and genetic susceptibility underpin this trend.

The hypothesis that the gut microbiome exerts a substantial impact on the development of immune-mediated diseases such as T1D has been proposed since the 1980s [16]. To investigate this hypothesis, researchers have performed experiments using germ-free rodents genetically predisposed to diabetes. These animals have been colonized with various microbial communities, administered probiotics, prebiotics, and antibiotics, fed diverse diets, and exposed to microbiota from different sources, providing insights into the microbiota’s role in disease development [17,18,19,20,21,22,23,24].

There exists a mutualistic relationship between humans and their gut flora that is essential for maintaining homeostasis [25]. Disruptions in the normal composition of these commensal communities, known as dysbiosis, can disturb this balance and contribute to the onset of various autoimmune diseases [26].

Among the potential pathogenic mechanisms involved in T1D, gut dysbiosis has been implicated in immune dysregulation and altered host–microbiota interactions. Evidence from the non-obese diabetic (NOD) mouse model supports the relevance of microbiota-related mechanisms in autoimmune diabetes development, although the causal sequence between microbial alterations and β-cell autoimmunity remains incompletely defined [27]. However, we acknowledge that it remains unclear whether these microbial changes are causal, reactive, or a combination of both in the development of T1D.

## 2. Implications of Microbiome Alterations in T1D Development and Progression

As we have previously noted, alterations in intestinal microflora composition occur prior to the onset of T1D. These changes are often characterized by decreased microbial diversity and an increase in Bacteroidetes among individuals with pre-T1D conditions. Notably, the abundance of *B. dorei* within the Bacteroidetes phylum has been associated with T1D risk in Finland and could serve as a potential predictor for the disease. Furthermore, these *Bacteroides* strains often exhibit resistance to common antibiotics and are linked to diets high in protein and fat [28].

The importance of assessing the advantages of using gut microbiome characteristics for predicting T1D, in comparison to the already established islet autoantibodies in clinical settings, is acknowledged. Evaluating the efficacy, specificity, and predictive value of gut microbiome components may offer valuable insights into their potential role as biomarkers, either alongside or as alternatives to traditional autoantibody testing.

Upon analyzing several case–control studies, including a previous assessment of gut microbial composition in 16 children diagnosed with T1D compared with 16 healthy controls, a significant decrease in the Firmicutes/Bacteroidetes (F/B) ratio was observed in individuals with T1D relative to those without the condition [29]. This finding is consistent with similar reductions in the F/B ratio reported across various studies. Indeed, recent investigations indicate that a diminished F/B ratio is a consistent feature of the intestinal microbiota profiles of T1D patients in both China and Turkey [30,31].

Moreover, longitudinal cohort studies have demonstrated a temporal decline in the F/B ratio in children as they progress from islet autoimmunity to the eventual development of autoimmune diabetes [32]. In tandem with this shift, we observed a decrease in the diversity of the gut microbiome, a phenomenon previously noted in children with established T1D [33,34], as well as in autoantibody-positive individuals [35]. Interestingly, this reduction in bacterial diversity was specifically linked to seroconverters who ultimately developed T1D, distinguishing them from those who did not progress to the disease state [36].

In addition to these overall trends, metagenomic analyses across multiple studies reveal a significant reduction in the presence of butyrate-producing bacteria, such as members of *Clostridium* clusters IV and XIVa, as well as mucin-degrading bacteria like *Prevotella* and *Akkermansia*, in individuals with T1D. For example, when comparing the gut microbiota of 28 children recently diagnosed with T1D against 27 age-matched controls, a higher prevalence of butyrate-producing species within *Clostridium* clusters IV and XIVa among healthy children relative to their T1D counterparts was found [36]. Moreover, lower levels of butyrate-producing bacteria have also been documented in adults with longstanding T1D as well as in children exhibiting pancreatic autoimmunity [35,36,37].

Complementing the observed decline in butyrate-producing bacteria—which are known to promote mucin synthesis—we noted that *Prevotella* and *Akkermansia*—potential indicators of increased mucin production—were significantly less abundant in seropositive individuals compared to healthy children, a finding consistent with the work of Brown and colleagues [35]. Comparative analysis of bacterial composition in children with T1D, those with maturity-onset diabetes of the young 2 (MODY2), and healthy controls using 16S rRNA gene sequencing methods revealed that children with T1D had reduced levels of *Bifidobacterium*. Notably, the microbiota composition in T1D patients differed not only from that of healthy subjects but also from individuals with MODY2 [33]. Furthermore, decreased levels of Bifidobacterium in T1D patients have been confirmed through multiple approaches, including microbial proteome analysis and stool cultures [38]. Finally, colonization by the intestinal fungus Candida albicans has been positively correlated with T1D development [39].

## 3. Interconnections Between Dysbiosis and Intestinal Permeability in Type 1 Diabetes

The interconnection between gut dysbiosis and intestinal permeability provides an important mechanistic framework for understanding how microbial alterations may contribute to autoimmune activation in T1D. Beyond broad changes in microbial diversity or phylum-level composition, recent evidence suggests that gut barrier dysfunction in T1D involves multiple structural and immunological layers, including epithelial tight junctions, the mucus barrier, antimicrobial peptides, microbial metabolites, and mucosal immune-cell activation. A prospective cohort study in children with islet autoimmunity and T1D showed that gut microbiome dysbiosis is associated with increased intestinal permeability, supporting the hypothesis that barrier dysfunction may occur during the preclinical or early clinical phases of disease progression [40].

At the epithelial level, altered regulation of tight-junction permeability has been linked to T1D susceptibility. Increased zonulin expression has been associated with enhanced gut permeability in individuals with T1D and their relatives, suggesting that impaired tight-junction regulation may represent a relevant barrier-related mechanism in genetically susceptible subjects [41]. More recent human data extend this concept by showing that the intestinal mucus layer is also altered in T1D. Mucus-layer abnormalities, changes in mucin and antimicrobial peptide expression, and dysbiosis of mucus-associated microbial communities may reduce the separation between luminal microorganisms and the epithelium, thereby increasing microbial contact with mucosal immune cells. These alterations have been associated with immune dysregulation in human T1D, including pro-inflammatory mucosal immune responses [42].

Functional microbiome alterations may further contribute to barrier instability. In children with new-onset T1D, integrative metagenomic and metabolomic analyses identified decreased butyrate production and bile acid metabolism, together with increased lipopolysaccharide biosynthesis at the species, gene, and metabolite levels [43]. These findings are relevant because microbial metabolites can influence epithelial integrity and mucosal immune tone, while microbial products such as lipopolysaccharide may amplify innate immune activation after crossing a compromised barrier. Experimental evidence provides additional mechanistic support, showing that loss of gut barrier integrity can activate islet-reactive T cells within the intestinal mucosa and trigger autoimmune diabetes under microbiota-dependent conditions [24,27].

Taken together, these findings suggest that dysbiosis and intestinal permeability may interact through a self-reinforcing loop: microbial imbalance alters barrier-supporting metabolites and mucus-associated communities, while barrier disruption facilitates microbial antigen translocation and mucosal immune activation. However, although experimental studies support a causal role for barrier disruption in autoimmune diabetes, human studies remain limited by their observational nature. Longitudinal multi-omics studies beginning before seroconversion are therefore needed to determine whether intestinal barrier impairment acts as an initiating trigger, an early amplifier, or a downstream consequence of immune-metabolic changes in T1D.

## 4. Interconnections Between the Microbiome and the Immune System in T1D

Early alterations in the intestinal microbiome, particularly reduced microbial diversity and increased abundance of Bacteroidetes taxa such as *Bacteroides dorei*, have been observed before the clinical onset of T1D and may serve as potential predictors of T1D-associated autoimmunity [28]. Gastrointestinal microbiota, due to its constant interaction with immune cells, is often regarded as the largest immune organ [44]. Several studies have shown that dysregulation of the mucosal immune system can occur in non-obese diabetic (NOD) mice before the onset of diabetes [45].

The gastrointestinal microflora plays a crucial role in the development and maturation of the immune system, with early childhood representing a critical period for establishing immune tolerance [46]. The Hygiene Hypothesis posits that reduced microbial exposure during early life—resulting from advancements in medicine and sanitation—has contributed to the rising incidence of autoimmune diseases, including T1D. In support of this, NOD mice are more likely to develop diabetes when raised in sterile or hygienic environments, while early-life infections with various bacteria have been shown to prevent the onset of T1D [47,48]. In contrast, alterations in gut microbiota composition may result in insufficient immune education, potentially leading to the destruction of insulin-producing beta cells and the development of T1D in genetically susceptible individuals [49,50].

Another hypothesized mechanism that links microbial dysbiosis to T1D pathogenesis is increased intestinal permeability, often referred to as “leaky gut.” This condition may arise independently or in conjunction with immunological dysregulation [51]. Human studies and animal models alike show that barrier dysfunction is a characteristic of T1D [52,53,54]. Some researchers have suggested that hyperglycemia and insulitis may contribute to increased gut epithelial permeability in T1D [55]. However, there is also evidence suggesting that “leaky gut” may serve as a primary driver rather than simply a consequence, as increased intestinal permeability has been observed before the development of insulitis and T1D [56,57].

The genetic composition of the host can influence microbial composition and function, thereby affecting the activation of innate and adaptive immunity, as well as susceptibility to several diseases in both animal models and humans [58,59,60]. In mice, inherited disease susceptibility can be modulated through colonization with specific microbes, dietary modifications, or antibiotic administration, leading to outcomes that range from accelerated autoimmunity to complete disease prevention. Genes such as PTPN22, INS, CTLA4, and IL2RA have been linked to T1D [61]. Environmental factors including viral infections like rubella and enterovirus, along with dietary components and influences from the microbiota, contribute to the development of T1D. These insights have sparked further investigation into the potential role of the gut microbiome in the onset and progression of T1D [62].

During the initial stages of insulitis, various lymphocyte populations, including macrophages, are observed to infiltrate the pancreatic tissue. Pancreatic macrophages, situated within the vasculature of the islets, maintain close proximity to β cells [63]. Prior investigations have indicated that these macrophages internalize dense core granules and subsequently present islet antigens to islet-reactive CD4+ T cells, promoting the destruction of β cells [64]. Studies conducted by Ferris et al. showed that macrophages within the islets of NOD mice exist in an activated state, marked by increased responsiveness to stimulation and elevated expression of inflammatory mediators such as Cxcl9, Ccl5, and Cd40. Intriguingly, the administration of lipopolysaccharide (LPS), a constituent of the outer membrane of Gram-negative bacteria, elicited rapid inflammatory responses within the pancreas, suggesting that islet macrophages possess the capacity to detect signals originating from the gut microbiome and potentially accelerate the progression of T1D [63].

The gut microbiome harbors a diverse collection of pathogen-associated molecular patterns, including LPS, lipoproteins, peptidoglycan, and nucleic acids [65]. These microbial elements can activate diverse Toll-like receptors (TLRs), giving rise to both pro-diabetogenic and anti-diabetogenic immune responses. TLR signaling is mediated by adaptor proteins like MyD88, which facilitate cellular responses to LPS. It is important to note that NOD mice deficient in MyD88 and housed in specific pathogen-free conditions were protected from diabetes development. Conversely, T1D developed in germ-free MyD88-deficient NOD mice, which underscores that the protective benefit is reliant on signals from the gut microbiome [66,67,68].

**Figure 1:**
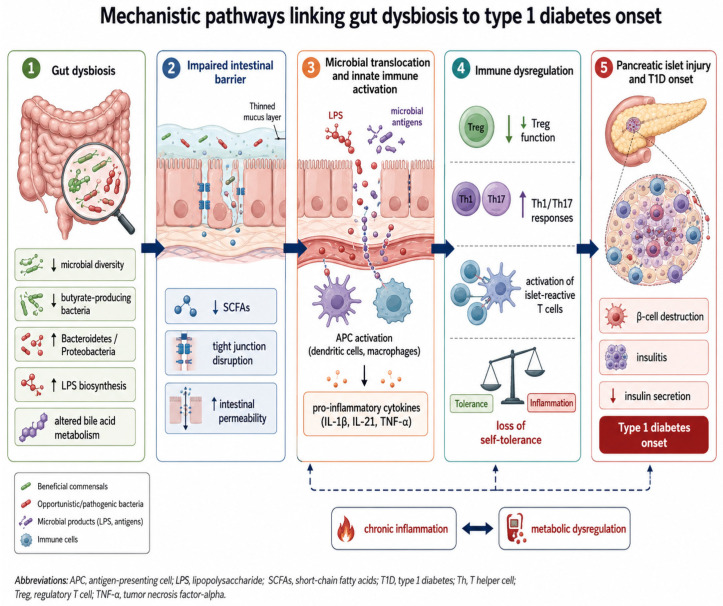
Mechanistic pathways linking gut dysbiosis to type 1 diabetes onset; ↓—low; ↑—high.

In addition to innate immune mechanisms, the interplay between gut microbes and adaptive immune responses appears to be integral to the development and progression of T1D. Specific gut bacteria influence the function and differentiation of T cell subsets; for instance, *Listeria* can induce a Th1 response, while segmented filamentous bacteria stimulate Th17 responses. Shifts in the composition of gut flora—including various *Clostridia* species—can enhance the generation of regulatory T cells (Tregs) [69,70]. Moreover, changes in microbiota composition may increase the population of type 1 regulatory T (Tr1) cells in the intestine, which can migrate to peripheral tissues, inhibiting the activation of effector T cells, and consequently reducing the incidence of diabetes [71] (Figure 1).

Nonetheless, the precise mechanisms by which these bacteria direct the differentiation of naïve T cells into specific helper T cell subsets remain largely undefined. One critical factor in this process is the production of SCFAs, which are produced by the fermentation of otherwise indigestible carbohydrates like cellulose—substrates that host enzymes cannot process [72]. SCFAs, such as acetate, propionate, and butyrate, play significant roles in preserving epithelial cell integrity and supporting the function of adaptive immune cells, such as enhancing the generation of Tregs in peripheral tissues [73,74]. Research by Mariño et al. has shown that a diet rich in acetate and butyrate protected mice from T1D development. They observed that acetate significantly reduced the frequency of autoreactive T cells in lymphoid tissues, while butyrate increased both the number and function of Tregs. Furthermore, these SCFAs helped to maintain intestinal integrity and reduced the levels of diabetogenic cytokines such as IL-21 in the serum [75].

*Bacteroides fragilis* is recognized as a key microbe due to its ability to induce Tregs and enhance their suppressive activity, a process dependent on the presence of butyrate [76,77]. Beyond modulation of T cell responses, SCFAs have been implicated in influencing B cell function as well [78]. Recent findings from Kim et al. show that SCFAs modulate the energy metabolism of B cells within the gut and promote their differentiation into plasma cells and memory B cells in response to antigens that activate B cell receptors, leading to an increased production of IgG and IgA [79,80]. Specifically, IgA plays a critical role in coating gut microbes, providing protection against pathogens, and maintaining intestinal homeostasis, especially during the early phases of microbiota colonization in newborns. As a result, IgA serves as a marker for gut microbiome colonization [79].

Furthermore, several bacterial proteins exhibit molecular structures resembling self-antigens found in the pancreas. For example, the Mgt protein from *Leptotrichia goodfellowii* shares similarity with the islet-specific glucose-6-phosphatase-related protein (IGRP), which is specifically expressed in pancreatic islets and belongs to the G6Pase family. The peptide IGRP206–214 (VYLKTNVFL) is a key epitope that activates diabetogenic NY8.3 T cells. Notably, a segment of the Mgt protein from *L. goodfellowii* shares sequence homology with IGRP206–214, and the Mgt267–275 fragment can activate NY8.3 T cells, promoting the development of T1D in NOD mice. Additional bacteria, such as *Flavobacteriia*, *Bacillus cereus*, and *Enterobacter mori* LMG 25706, also contain homologous peptides to IGRP206–214 that have demonstrated activity both in vitro and in vivo. These findings suggest that certain diabetogenic microbes in the gut may initiate or accelerate T1D onset through molecular mimicry mechanisms [81,82,83].

The concept of molecular mimicry holds significance not only for T1D but also for other autoimmune conditions, including systemic lupus erythematosus, characterized by extensive multi-system damage, where B cells and autoantibodies play a crucial role in disease progression. Research by Hevia et al., comparing 20 patients in remission with matched healthy controls, suggests that molecular mimicry does not always exacerbate autoimmune diseases. Specifically, Bacteroides species express an integrase containing a low-avidity mimotope for IGRP206-214, which facilitates the recruitment of diabetogenic CD8+ T cells to the gut, thus diminishing inflammation by targeting gut dendritic cells. Therefore, while molecular mimicry serves as a key mechanism through which the gut microbiome may initiate autoimmune diseases, its impact varies depending on the specific disease and context [84,85].

In a notable study, Bosi et al. assessed intestinal permeability in 81 individuals at various stages of T1D, along with a control group of 40 healthy individuals, concluding that increased gut permeability occurred prior to the onset of the disease [86]. Consistent with these findings, a recent investigation by Harbison and colleagues found that children at risk for T1D had increased intestinal permeability, which correlated with gut microbiota dysbiosis [40]. Furthermore, the administration of a hydrolyzed casein diet has been reported to protect against T1D by enhancing gut integrity in diabetes-prone-Bio Breeding rats [87].

Support for this concept comes from observations that infection with the barrier-disrupting *C. rodentium* accelerates insulitis in NOD mice, whereas NOD mice infected with the DeltaespF strain, which does not compromise gut integrity, did not develop insulitis [88]. Together, these findings strongly imply that a “leaky gut” may play a significant role in the progression of T1D and may represent an early event in the disease’s evolution.

It is hypothesized that increased intestinal permeability, resulting from a compromised gut barrier [89], may allow the unregulated passage of exogenous antigens, particularly microbial components [90]. Translocation of these elements into systemic circulation may induce systemic inflammation and facilitate autoimmune processes through direct damage to pancreatic beta cells [91]. Alternatively, microbial components may be taken up by antigen-presenting cells (APCs), which process and present these antigens to autoreactive T cells, ultimately leading to the destruction of islet beta cells [92].

Another proposed mechanism through which microbial antigens trigger diabetes is molecular mimicry. Certain microbial antigens share structural similarities with islet self-antigens, which may induce T cell cross-reactivity and autoimmune attack on beta cells [93]. Indeed, a recent study indicated that oral administration of *Bacteroides fragilis* in the setting of chemically induced gut barrier compromise resulted in rapid disease progression in NOD mice, emphasizing the role of microbial translocation in advancing T1D [94].

## 5. Microbiota Implications in T1D

In T1D, the gut microbiota is characterized by reduced production of butyrate, disrupted bile acid metabolism, and increased synthesis of lipopolysaccharide (LPS) at the levels of microbial species, genes, and metabolites. These alterations are not merely associative but appear to play a functional role in metabolic dysregulation. For instance, studies in antibiotic-treated mice have demonstrated that microbiota perturbations can increase fasting glucose levels and impair insulin sensitivity. In streptozotocin-induced T1D models, butyrate exerts protective effects by preserving islet architecture and function, whereas LPS aggravates pancreatic inflammation and immune activation. Mechanistically, butyrate has been shown to upregulate the expression of *Insulin1* and *Insulin2*, highlighting the divergent and biologically significant roles of these microbial metabolites in T1D pathophysiology [43].

Over the past three decades, the global incidence of T1D has risen markedly [95], with the steepest increases observed in rapidly developing regions such as China. This trend underscores the growing importance of environmental and lifestyle-related factors in disease etiology [96,97]. T1D is now widely recognized as a multifactorial condition resulting from complex interactions between genetic susceptibility and environmental exposures, including viral infections, dietary patterns, antibiotic use, and shifts in gut microbiota composition [98].

A consistent feature of T1D-associated dysbiosis is reduced microbial diversity, often accompanied by alterations in the Firmicutes/Bacteroidetes (F/B) ratio [20,29] and a depletion of short-chain fatty acid (SCFA)-producing bacteria [99,100]. Functional insights from the TEDDY study revealed that, despite minimal differences in overall microbial composition between seroconverted children and controls, there was a significant reduction in microbial genes involved in fermentation and SCFA production [101]. This suggests that functional capacity, rather than taxonomic composition alone, may be critical in disease development. Supporting this, *Bacteroides stercoris* has been found to be more abundant in Italian children with T1D and is associated with disruptions in multiple metabolic pathways [23]. Additional studies have consistently reported increased LPS biosynthesis, reduced butyrate production, and altered bile acid profiles in individuals with new-onset T1D. Collectively, these findings indicate that specific microbial configurations and metabolite profiles may serve as early biomarkers or predictors of disease onset. Integrative multi-omics approaches, combining metagenomics and metaproteomics, have further demonstrated correlations between microbial signatures and host proteomic traits in T1D populations [102].

Attempts to prevent T1D through dietary or pharmacological interventions have yielded limited success. Strategies such as early weaning to hydrolyzed formulas or delayed gluten introduction have not significantly reduced disease incidence [103,104]. Similarly, interventions aimed at preserving β-cell function—such as oral antigen administration or nicotinamide supplementation—have not demonstrated efficacy [105,106]. However, emerging evidence suggests that early-life exposure to probiotics may confer protective effects in genetically predisposed individuals, particularly those carrying the HLA DR3/4 genotype [107].

The establishment of the microbiota begins prenatally and is influenced by both maternal and paternal factors [108]. Maternal infections can facilitate microbial transfer to the fetus, while the mode of delivery plays a critical role in shaping neonatal colonization patterns. Vaginally delivered infants acquire microbiota resembling the maternal vaginal community, whereas those born via cesarean section are colonized predominantly by skin- and environment-associated microbes [109]. Beyond the gut, the oral microbiota—comprising approximately 700 species—represents another significant microbial ecosystem with potential systemic implications [110].

Recent interventional studies have explored microbiome modulation in children with T1D. Clinical trials using probiotics such as *Lactobacillus rhamnosus* GG and *Bifidobacterium lactis* Bb12, as well as prebiotic supplementation with oligofructose-enriched inulin, have shown promising but preliminary results [111,112]. Notably, children who progress to T1D tend to exhibit increased abundance of taxa such as *Streptococcus thermophilus*, *Lactococcus lactis*, *Bifidobacterium pseudocatenulatum*, *Roseburia hominis*, and *Alistipes shahii*. In contrast, healthy controls display enrichment in genes associated with fermentation and SCFA biosynthesis, reinforcing the importance of microbial metabolic function in disease protection [101].

Emerging data also indicate that T1D is associated with compromised mucosal barrier integrity and impaired pancreatic exocrine function [38,102]. Persistent dysbiosis may contribute to increased intestinal permeability, facilitating the translocation of microbial components such as LPS into systemic circulation. This, in turn, can promote chronic inflammation and disrupt immune tolerance. In individuals with long-standing T1D, microbiome alterations have been linked to glycemic control and the development of complications [113]. Experimental evidence further suggests that maternal microbiota composition can influence disease susceptibility, as demonstrated by the protective effects of maternal cecal microbiota in antibiotic-treated NOD mice [114]. Disruption of gut barrier function may also activate islet-specific T cells, thereby contributing to autoimmune β-cell destruction [91].

To better understand these mechanisms, integrative metagenomic and metabolomic analyses have been conducted in pediatric patients with new-onset T1D. These studies aim to identify key microbial taxa, metabolic pathways, and fecal metabolites associated with disease risk. Complementary approaches, including fecal microbiota transplantation (FMT) and animal models, have provided further insights into host–microbiota interactions during disease progression [43].

Clinical and microbiome comparisons between children with T1D and healthy controls reveal distinct differences. T1D patients typically present with lower body mass index, poorer glycemic control, reduced C-peptide levels, elevated inflammatory markers, decreased HDL cholesterol, and increased triglycerides. Microbiome analyses demonstrate reduced richness and diversity, as reflected by lower Chao1 and Shannon indices, along with compositional shifts characterized by decreased Firmicutes and increased Bacteroidetes and Proteobacteria. At the genus level, T1D is consistently associated with a reduction in butyrate-producing bacteria and an enrichment of opportunistic pathogens (Table 1) [43].

At the species level, beneficial microbes such as *Faecalibacterium prausnitzii* are depleted, while potentially pathogenic species like *Escherichia coli* are enriched. Functional profiling reveals reduced gene counts, decreased abundance of carbohydrate-active enzymes, and impaired pathways related to carbohydrate metabolism, alongside increased LPS biosynthesis. Metabolomic analyses further show decreased levels of SCFAs—particularly butyrate and acetate—as well as reduced bile acids and GLP-1. Conversely, markers of inflammation and gut-derived antigen load, including FGF19, LPS-binding protein, and IL-1β, are elevated. Importantly, T1D-associated metabolites correlate positively with HbA1c and fasting glucose levels, whereas butyrate-related metabolites show positive associations with beneficial taxa and negative associations with opportunistic pathogens [43].

Although β-cell destruction in T1D is primarily driven by autoimmune processes involving specific autoantibodies, these markers are absent in 10–30% of cases, indicating heterogeneity in disease mechanisms [115]. While experimental models strongly support a role for the microbiome in T1D pathogenesis, translating these findings to humans remains challenging due to the complexity of host–microbiota interactions and the scarcity of long-term controlled studies [116].

T1D typically manifests in early childhood, a period characterized by rapid and dynamic changes in gut microbiota influenced by diet, infections, and environmental exposures. Dysbiosis during this critical window may impair immune tolerance and contribute to disease onset, potentially through increased intestinal permeability [51]. Certain gut bacteria produce glutamic acid decarboxylase (GAD), which may trigger autoimmune responses due to molecular mimicry with human GAD65 [117]. Elevated circulating LPS levels have also been observed in T1D patients [118], and LPS derived from specific *Bacteroides* species may modulate immune responses and increase susceptibility [119]. Through activation of Toll-like receptor 4 (TLR4), LPS stimulates pro-inflammatory cytokine production, thereby contributing to chronic inflammation and β-cell damage [120].

Reduced microbial diversity is a hallmark of T1D, affecting both alpha- and beta-diversity as well as overall richness [121,122]. This reduction often precedes clinical onset and is more pronounced in autoantibody-positive individuals, suggesting its potential as an early biomarker of disease progression [123,124]. During the transition from seroconversion to overt disease, increases in pro-inflammatory metabolic pathways occur alongside declining microbial diversity, further supporting a causal link between microbiome alterations and T1D [125].

Alterations in the Firmicutes/Bacteroidetes ratio are frequently reported in T1D, with a general trend toward reduction in affected or at-risk individuals. This ratio also appears to correlate with autoantibody burden [126]. Given that Firmicutes are major producers of butyrate and Bacteroidetes contribute to LPS synthesis, shifts in this balance may underlie key metabolic and immunological disturbances observed in T1D [127]. However, inconsistencies across studies indicate that this relationship is complex and requires further investigation [128].

At the taxonomic level, T1D is often associated with increased abundance of genera such as *Bacteroides*, *Clostridium*, and *Lactobacillus*, while healthy individuals tend to have higher levels of *Prevotella*, *Akkermansia*, and other butyrate-producing taxa. Notably, *Bacteroides dorei* and *Bacteroides vulgatus* are enriched in seroconverted individuals, with *B. dorei* showing peaks prior to autoantibody detection, suggesting a potential predictive role for these microbes in high-risk populations [127,129].

SCFAs—including acetate, propionate, and butyrate—are key metabolites produced through microbial fermentation of dietary fibers and play crucial roles in immune regulation and metabolic homeostasis. Individuals with T1D consistently exhibit lower levels of these metabolites, particularly butyrate. This deficiency may compromise gut barrier integrity and reduce the differentiation of regulatory T cells, thereby impairing immune tolerance and facilitating autoimmune destruction of pancreatic β cells [130,131].

## 6. Blood and Oral Microbiome Connection as a Prognostic Indicator for T1D Complications

Traditionally, in healthy organisms, blood is considered sterile due to the lack of active microbial growth. However, advancements in DNA sequencing have uncovered the presence of live microorganisms or bacterial DNA in both healthy individuals and those suffering from non-communicable diseases such as type 2 diabetes (T2D) [132], liver fibrosis [133,134,135], rheumatoid arthritis [136], cancer [137,138], cardiovascular diseases [139], Parkinson’s disease [140], and various immune disorders [141]. The microorganisms in the bloodstream are thought to originate from other body areas, primarily the gut, mouth, and skin, and their prevalence is associated with intestinal permeability [142,143,144].

Emerging research indicates that the blood microbiome may contribute to chronic metabolic diseases, including T2D, cardiovascular conditions, and inflammatory disorders [145]. The presence of bacteria in the bloodstream can provoke immune responses, leading to questions regarding the role of the blood microbiome in diseases such as T1D; however, no direct studies on this have been conducted. Notably, research has indicated that individuals with T2D exhibit an imbalance in gut microbiota and increased blood levels of certain bacteria. Sato et al. [146] focus on characterizing the blood microbiome in children newly diagnosed with T1D and compare it to the gut and oral microbiomes to highlight potential microbial connections. The study recruited children with newly diagnosed T1D and matched non-diabetic controls, analyzing their blood samples through 16S rRNA gene sequencing. Results showed that the blood microbiome of T1D patients exhibited increased microbial diversity compared to controls, with a notable prevalence of the *Proteobacteria phylum*. Specific genera found enriched in the T1D patients included *Cupriavidus* and *Sphingomonas*, while others were diminished. Also, the blood microbiome exhibited distinct characteristics from the gut and oral microbiomes, indicating the role of barrier systems in limiting bacterial translocation [146]. The analysis also revealed that certain microbial genera were significantly correlated with metabolic markers in T1D patients, suggesting strong associations between the microbiome and systemic inflammation. The study highlights the complexity of the blood microbiome in T1D and its potential role as a biomarker and therapeutic target.

Emerging evidence highlights an interrelationship between the oral microbiome and systemic diseases, including T1D [147]. The onset of damage to β cells may occur years in advance of the presentation of clinical symptoms, thus raising the possibility that analysis of the oral microbiota could enable earlier diagnosis and therapeutic intervention for T1D in children with β cell autoimmunity [148]. Individuals with T1D frequently experience dry mouth, oral acidosis, and periodontal disease, all of which are associated with poor glycemic control [149,150].

As a consequence of hyperglycemia-driven dehydration, reduced salivary flow may disrupt the balance of the oral microbiome, allowing for the proliferation of pathogenic bacteria such as *Streptococcus mutans* and *Lactobacillus*, ultimately leading to oral health complications [151]. Periodontal disease is a common comorbidity in T1D and is linked to chronic inflammation elicited by bacteria such as *Porphyromonas gingivalis*, which trigger immune responses that can damage gingival tissue [152]. Elevated glucose levels in saliva promote oral dysbiosis, shifting the bacterial landscape toward acidogenic species that exacerbate dental decay [153]. A Dutch study found that T1D children had higher abundances of Actinobacteria and Firmicutes compared to healthy controls, with a notable increase in Streptococcus species linked to poor glycemic control [154]. Oral microbiota dysbiosis during acute T1D could be partially improved with glycemic control, otherwise, it was characterized by reduced diversity and increased opportunistic pathogens [155]. Emerging research indicates that gut and oral dysbiosis may mutually influence each other and systemic health. For example, shared microbial species have been identified in both sites, and oral community profiles can predict gut microbiota compositions [156]. In T1D patients, both oral and gut dysbiosis are associated with poor glycemic control and systemic inflammation, suggesting possible translocation of bacteria through the oral-gut axis, which could contribute to systemic disease development [157,158].

Our findings, combined with those of other researchers, suggest the existence of interactions between the oral and gut microbiota in the context of T1D. As an example, a study by Yuan et al. analyzed the microbiome composition in blood, oral, and gut samples from 64 children diagnosed with T1D and 77 control subjects, revealing a partial overlap between the blood microbiome and the microbial communities present in both the oral cavity and gut. This observation prompted the suggestion that bacteria may translocate from the gut and mouth into the bloodstream, with T1D patients exhibiting elevated levels of pathogenic bacteria and higher concentrations of inflammatory markers [159]. Consistent with these findings, Wang et al. identified correlations between oropharyngeal and gut microbiota in a cohort of 13 children with T1D, indicating that populations of pathogenic bacteria at both locations may fluctuate together, with notable relationships observed between oropharyngeal *Staphylococcus* and intestinal *Ruminococcaceae*, as well as a positive correlation with blood C-peptide levels [160].

Furthermore, the work of Kunath et al. has demonstrated that alterations in the oral microbiome can affect the composition of microbial communities residing in the gut and promote inflammation. Specifically, they found that a decrease in *S. salivarius* in the mouth was associated with reduced bacterial diversity in the gut and an increase in pathogenic *Enterobacteriaceae*. They did not, however, identify direct evidence for the mechanisms controlling the transfer of microbiota from the mouth to the gut, suggesting that factors such as salivary flow and pH may influence this process [161].

In summary, the available evidence supports the existence of notable interactions between oral and gut microbiota in T1DM, although additional investigations are necessary. A more complete understanding of these mechanisms may lead to the development of innovative therapeutic strategies for managing, and potentially preventing, this complex condition.

## 7. Insights into the Interplay Between the Gut Virome, Mycobiome, and T1D Development

Currently, there are limited clinical studies exploring the role of the intestinal virome in the development of T1D in humans [162,163,164]. In one such study, stool samples from 19 children with early advanced islet autoimmunity—who subsequently progressed to clinical T1D—along with closely matched controls, were analyzed using next-generation sequencing methods. The analyses revealed no significant alterations in the intestinal virome prior to the appearance of T1D-associated autoantibodies [162]. In a subsequent report from the same DIPP Study cohort, associations between islet autoimmunity and both the intestinal bacteriome and virome were assessed. The results indicated that four specific bacterial operational taxonomic units were less abundant in children progressing toward islet autoimmunity. Furthermore, a quantitative relationship between *CrAssphage* and *Bacteroides* species suggested that *CrAssphage* may contribute to the dysbiosis associated with islet autoimmunity [163].

In a longitudinal study of Finnish and Estonian children, healthy controls were found to have a more diverse intestinal virome, with a notably higher abundance of Circoviridae-related sequences compared to children exhibiting T1D-associated autoantibodies [164]. Although a variety of viral sequences, including enteroviruses and rotaviruses, were identified in T1D group, none showed a direct association with islet autoimmunity. Importantly, bacteriophage contigs linked to T1D demonstrated significant correlations with specific elements of the bacterial microbiome, underscoring the dynamic interplay between viral and bacterial communities. Overall, the findings suggest that certain viral signatures may represent typical intestinal development and could potentially act as indicators of future microbiome shifts [164].

In the future, more extensive studies could determine whether alterations in the intestinal virome precede changes in bacterial composition related to islet autoimmunity or whether they occur independently. Given that bacteriophages influence bacterial populations, shifts in their abundance and diversity may disrupt intestinal homeostasis and promote dysbiosis [165]. In addition, changes in fungal communities, such as overgrowth of *Candida* species, have been observed in individuals with both T1D and inflammatory bowel diseases. It is possible that poor glycemic control could facilitate fungal overgrowth, increasing susceptibility to recurrent and prolonged fungal infections in these patients [166].

## 8. Longitudinal Changes in Gut Microbiota During Type 1 Diabetes Treatment

Beyond disease prediction, controlling the gut microbiome for T1D treatment appears promising. Animal studies have demonstrated that the incidence of T1D in genetically susceptible mice significantly decreased when housed with healthy mice or administered fecal samples via oral gavage from non-diabetic donors. Additionally, oral probiotic treatments have been shown to prevent the onset of diabetes in NOD mice [167].

Furthermore, recent research indicates that precise modulation of the gut microbiome—such as using tungstate to inhibit molybdenum-cofactor-dependent microbial respiration pathways—can alleviate inflammatory bowel disease with minimal side effects [168]. This suggests that microbiome engineering or targeted editing could become a viable therapeutic approach for T1D in the future. Therefore, microbiome modulation offers a promising avenue for developing novel treatments.

However, it is important to consider that current T1D therapies, including various drug treatments, may influence the composition and function of the gut microbiome. Currently, insulin remains the primary standard for managing glycemic levels in patients with T1D, while immunomodulators and traditional Chinese medicine approaches are also under evaluation in preclinical and clinical studies [59].

### 8.1. Insulin

Previous research has indicated that several medications commonly used in the treatment of T2D, such as metformin, liraglutide, and saxagliptin, may alter the composition of the gut microbiome [169,170]. Despite insulin therapy being the primary intervention for glycemic management in T1D, its specific effects on the gut microbiome remain under investigation and warrant further study.

In a recent investigation [171], the effects of insulin administration on the gut microbiome using the NOD mouse model were examined. From 4 to 9 weeks of age, the mice received oral porcine insulin via gavage twice per week, followed by weekly doses for a total duration of 21 weeks. Fecal samples were collected, and the composition of the gut microflora was analyzed using 16S rRNA sequencing. The findings indicated that oral insulin administration did not significantly affect gut microbiota [171]. One possible explanation for this lack of impact is that most of the insulin is degraded during its passage through the intestine, leading to low bioavailability.

### 8.2. Immunomodulators

Since T1D is an autoimmune disease, various immunomodulatory therapies have been investigated for potential use in disease management. These include rituximab, an anti-CD20 chimeric monoclonal antibody that targets and depletes B cells; humanized anti-CD3 monoclonal antibodies, such as teplizumab and otelixizumab; GAD65 designed to induce antigen-specific immune tolerance; DiaPep277, a peptide originating from human heat shock protein 60; and abatacept, a CTLA-4-Ig fusion protein that inhibits T cell activation. Despite the modest effectiveness of most of these agents in clinical trials, their effects on the gut microbiome remain uninvestigated [172,173,174,175,176,177].

### 8.3. Traditional Chinese Medicine

Given that most herbal medicines (HMs) are administered orally, it is understood that they may induce specific alterations in the gut microflora. A reciprocal interaction seems to occur between HMs and gut microbes: gut bacteria play a critical role in HM therapy by biotransforming HM compounds into smaller, more bioavailable forms, while HM chemicals can also modulate the composition of the intestinal microbiota and ameliorate pathological conditions [59].

Currently, most HMs have not been carefully studied in the context of T1D, with only a few notable exceptions. For instance, Ganoderma lucidum has been shown to normalize an elevated F/B ratio. Berberine, a benzylisoquinoline alkaloid extracted from plants, has demonstrated protective effects in NOD mice by modulating immune responses [178]. Additionally, artemisinin and Danzhi Jiangtang Capsule have been reported to improve β cell function by increasing β-cell total mass and decreasing pancreatic β-cell apoptosis respectively [179,180]. However, the influence of HMs on the gut microbiome and the mechanisms through which they may mediate disease protection are still not fully understood.

### 8.4. Long-Term Outcomes of Gut Microbiome Modulation Strategies in T1D Treatment

Modulating the microbiome represents a promising avenue for enhancing health outcomes in diabetic patients, particularly those with T1D [181]. Therapeutic strategies centered on nutrition aim to improve gut microbiota composition, support glycemic control, reduce inflammation, and promote overall well-being in children diagnosed with T1D. Emerging interventions, such as FMT, have demonstrated positive effects in correcting microbial dysbiosis and restoring immune balance across a range of diseases, including T1D [182].

Supplementation with prebiotics, probiotics, and synbiotics may help to reverse microbial imbalances in children with T1D by promoting beneficial bacteria and decreasing pathogenic species, thereby reducing intestinal permeability and inflammation [183]. For example, prebiotics like human milk oligosaccharides and high amylose maize starch have been shown to increase beneficial bacterial populations, improve glycemic control, and support β-cell function [184,185]. Pilot studies suggest that prebiotic supplementation can lead to increased plasma C-peptide levels, improved intestinal barrier function, and higher *Bifidobacteria abundance* [186].

While some evidence supports the potential of *Lactobacillus* and *Bifidobacterium strains* in diabetes management, research specific to children with T1D remains limited and yields inconsistent results regarding improvements in glycemic markers and inflammatory parameters [187,188]. Notably, one study found that a multi-strain probiotic formulation could enhance glycemic control and reduce insulin requirements in newly diagnosed T1D children [189,190]. Conversely, other trials have reported no significant benefit of specific probiotic strains in preserving pancreatic β-cell function during early disease stages [191]. Additionally, synbiotics—product combinations of probiotics and prebiotics—show promise in alleviating gut dysbiosis, improving glycemic status, promoting SCFA production, and lowering inflammatory cytokines, thereby strengthening gut barrier integrity [192,193].

Interestingly, supplementation with butyrate has been shown to reduce elevated fasting blood glucose levels in FMT-treated T1D models and significantly improve insulin levels. However, we observed no significant differences between the treatment groups in oral glucose tolerance tests, insulin and C-peptide levels, or pancreatic tissue histology. Moreover, T1D mice that received FMT exhibited a decreased *Firmicutes*/*Bacteroidetes* ratio and lower abundance of butyrate-producing bacteria, specifically Faecalibaculum, when compared to controls. These observations suggest that butyrate’s role in modulating immune responses presents potential therapeutic strategies that could be implemented through dietary interventions, although further investigations are needed to confirm their safety and effectiveness in humans [43].

Despite these encouraging results, studies on the protective effects of probiotics against T1D in humans remain limited. For instance, a significant study by Uusitalo et al. [107] demonstrated that early probiotic supplementation during the first four weeks of life reduced the risk of developing beta cell autoimmunity in genetically predisposed infants. More recently, a single-center, randomized, double-blind, placebo-controlled pilot trial involving children with T1D, conducted for at least one year found that prebiotic intake could alter gut microbiota composition and reduce intestinal permeability, which was associated with improved beta cell function. However, this study did not demonstrate significant improvements in glycemic control, possibly due to the small size of the study cohort and the limited duration of the intervention [186]. In addition, Groele et al. are currently conducting a double-blind, randomized, placebo-controlled study designed to assess whether probiotics can improve beta cell function by modulating immune responses in children recently diagnosed with T1D [191].

## 9. Conclusions

This narrative review highlights the indispensable role of the intestinal microbiota in the pathogenesis of T1D, a multifaceted autoimmune disorder characterized by the selective destruction of pancreatic β cells. Intestinal dysbiosis, a recurrent hallmark across disparate investigations, manifests as a confluence of diminished microbial diversity, perturbations in the ratios of predominant bacterial phyla (Firmicutes and Bacteroidetes), and quantitative/qualitative aberrations in the generation of key bacterial-derived metabolites. These microbial alterations may contribute to impaired intestinal barrier integrity, thereby facilitating the translocation of microbial components and luminal antigens that can activate islet-reactive T cells and promote autoimmune responses involved in β-cell destruction. The complex, dynamic interplay between the composition and function of the gut microbiome, the host’s predisposing genetic architecture, and the intricate regulation of the immune system’s effector and regulatory arms, highlights the therapeutic potential of targeted interventions designed to modulate the intestinal microbiota.

Future research should prioritize several key directions. Foremost among these is the need for long-term, longitudinal cohort studies beginning early in ontogeny, which are essential for clarifying the temporal interplay between microbial changes, the maturation of immune responses, and the eventual development of T1D. Such investigations must employ advanced computational techniques to model the complex interplay between microbial community structure, function, and host phenotype. Second, mechanistic studies are needed to deconstruct the molecular pathways through which specific microbial taxa and/or their cognate metabolites influence the development and function of tolerogenic immune cells, the integrity of the intestinal epithelial barrier, and the viability/functionality of pancreatic β cells. These investigations will necessitate the utilization of gnotobiotic animal models, coupled with sophisticated in vitro co-culture systems, to investigate the biochemical and cellular mechanisms governing host-microbe interactions. Third, systems biology approaches that integrate metagenomic, metatranscriptomic, metabolomic, proteomic, and immunomic data will provide a holistic, network-level understanding of the complex interplay between microbial communities and host immune responses. These multi-omic datasets must be analyzed using sophisticated bioinformatic pipelines to identify predictive biomarkers of disease risk and progression. Fourth, rigorously designed, double-blind, placebo-controlled clinical trials are warranted to evaluate the safety and efficacy of microbiome-targeted therapies for the prevention and/or treatment of T1D. Such trials should employ personalized therapeutic strategies, guided by individual microbiome profiles, genetic risk scores, and immunological signatures. Finally, future research should extend its scope to encompass the currently under-explored contributions of the intestinal virome and mycobiome to T1D pathogenesis, and delineate the nature of their interactions with the bacterial microbiome and host immune responses.

In conclusion, the microbiome may influence the development of autoimmune diseases through its central role in immune system maturation, maintenance of intestinal barrier integrity, regulation of inflammatory responses, and preservation of immune tolerance. The gut microbiota and the immune system are closely interconnected in a bidirectional relationship: microbial communities contribute to the development of regulatory T cells, support mucosal immune balance, and produce metabolites such as short-chain fatty acids, particularly butyrate, which strengthen epithelial tight junctions and modulate both innate and adaptive immune responses. When dysbiosis occurs, this balance may be disrupted through reduced beneficial bacteria, decreased SCFA production, increased abundance of LPS-producing taxa, and impaired barrier function. These changes can facilitate microbial antigen translocation, activate antigen-presenting cells, stimulate pro-inflammatory pathways such as Th1 and Th17 responses, and contribute to the loss of self-tolerance and activation of autoreactive immune cells, mechanisms that are highly relevant in autoimmune diseases such as type 1 diabetes. However, long-term microbiome research remains challenging because microbial communities are highly dynamic and influenced by age, diet, genetics, medication and antibiotic exposure, infections, geography, lifestyle, and environmental factors. These variables make it difficult to determine whether microbiome alterations are a cause of autoimmune disease, a consequence of early immune-metabolic changes, or part of a bidirectional pathogenic process. Additional difficulties include the need for large cohorts, repeated sample collection, standardized sequencing and bioinformatic methods, careful control of confounders, and long follow-up periods beginning before clinical disease onset. Although microbiome-based therapies represent a promising field, current evidence does not support their ability to completely prevent type 1 diabetes. Rather, interventions such as probiotics, prebiotics, dietary modulation, or other microbiome-targeted strategies should currently be regarded as adjunctive or preventive-support approaches that may help reduce disease risk, delay onset, support intestinal barrier integrity, and modulate immune responses in selected individuals. In this context, personalized therapy is particularly important, as patients differ in genetic susceptibility, immune profile, microbiome composition, environmental exposures, disease stage, and treatment response. Tailoring interventions to each patient’s biological and clinical characteristics may improve therapeutic efficacy, reduce unnecessary interventions and adverse effects, preserve immune tolerance, and support better long-term disease control. If validated through rigorous longitudinal studies and clinical trials, microbiome-targeted therapies could contribute to improved glycemic stability, reduced inflammatory burden, preservation of residual β-cell function, reduced treatment burden, and ultimately better quality of life for patients at risk for, or living with, autoimmune diseases.

## Figures and Tables

**Table 1:** A synthesized form of the presented studies.

Aspect	Findings	Conclusions
Study Population	Pediatric patients with new-onset T1D vs. non-diabetic controls [43]	Focused on early-stage T1D; comparison of clinical, microbiota, metabolite profiles [43]
Clinical Characteristics	Lower BMI, poor glycemic control, lower C-peptide; elevated inflammatory markers (WBC, neutrophils) [43]	T1D associated with metabolic dysregulation and systemic inflammation [43]
Lipid Profile	Reduced HDL-C, increased triglycerides [43]	Lipid metabolism disruption linked to T1D [43]
Gut Microbiota Diversity & Structure	Lower richness/diversity (Chao 1, Shannon indices); distinct microbial communities (PCoA)	Microbial dysbiosis in T1D; decreased diversity and altered community structure [20,29,43]
Microbial Composition	Decreased Firmicutes, increased Bacteroidetes & Proteobacteria; reduced butyrate producers (e.g., *Faecalibacterium prausnitzii*); increased opportunistic pathogens (e.g., *E. coli*) [20,24,29,43,100]	Microbial shifts favoring pathogenic inflammation and reduced beneficial metabolites
Functional Pathways	Lower carbohydrate-active enzymes; decreased starch and sucrose metabolism; increased LPS synthesis [23,43,101]	Functional impairments linked to gut inflammation and metabolic disturbances
Fecal Metabolites	Increased levels of 5 specific metabolites; decreased 21 metabolites including bile acids; lower SCFA levels (butyrate, acetate) [43,102]	Metabolic profile indicating diminished gut microbial activity and oxidative balance
Serum and Inflammatory Markers	Reduced GLP-1; elevated FGF19, LBP, IL-1β [43]	Enhanced gut-derived inflammation and metabolic hormone alteration
Correlations	Positive link between T1D-associated metabolites and HbA1c/glucose; butyrate metabolites correlated with beneficial species [43,102,113]	Microbial metabolites associated with glycemic control and microbial composition
Overall Conclusions	Gut microbiota dysbiosis (decreased diversity, butyrate producers, altered functional pathways) and associated metabolite changes are linked to T1D pathogenesis, inflammation, and metabolic disturbances in pediatric patients [20,24,29,43,100,101,102]	Targeting gut microbiota composition and function may offer new avenues for early intervention and understanding T1D mechanisms

## Data Availability

No new data were created or analyzed in this study. Data sharing is not applicable to this article.
